# Neurocutaneous Melanosis in Children and Adults With Clinical, Radiological, and Pathogenic Evaluation: Case Report and Review of the Literature

**DOI:** 10.1002/ccr3.71365

**Published:** 2025-10-25

**Authors:** Yinghua Ji, Keya Zhi, Han Yang, Xiangli Meng, Jin Wang, Jinling Xie, Yong Tan, Ping Lu, Yana Zhang

**Affiliations:** ^1^ Department of Oncology The First Affiliated Hospital of Xinxiang Medical University Xinxiang China; ^2^ Department of Life Science Research Center The First Affiliated Hospital of Xinxiang Medical University Xinxiang China; ^3^ Department of Gastrointestinal Surgery The First Affiliated Hospital of Xinxiang Medical University Xinxiang China

**Keywords:** diagnosis, melanocytic nevi, melanoma, neurocutaneous melanosis, therapy

## Abstract

Neurocutaneous melanosis (NCM) is a rare, non‐inherited disease characterized by the presence of giant congenital melanocytic nevi (CMN) or multiple CMN, and it is often associated with meningeal melanosis or meningeal melanoma. This report presents two representative cases of NCM: one involving a 6‐year‐old female patient and the other involving a 30‐year‐old male patient. Both patients were born with severe CMN, including lesions on their hairy trunk, and later acquired malignant leptomeningeal involvement. We evaluated the clinical, radiological, and pathological features of cutaneous and central nervous system (CNS) lesions in these two patients, emphasizing their similarities and distinct manifestations. The research analysis highlights that individualized monitoring and related intervention strategies may improve the clinical prognosis of patients with this uncommon and potentially fatal disease through the process of early identification of high‐risk features associated with malignant transformation.

**Summary**

- Neurocutaneous melanosis is a rare neurocutaneous disorder in which delayed intervention may increase the risk of malignant transformation.
- For children or adults with a history of extensive cutaneous melanosis and concurrent neurological symptoms, NCM should be considered during the differential diagnosis.

## 1 Introduction

Neurocutaneous melanosis (NCM) is a rare neurocutaneous disorder characterized by the abnormal proliferation and deposition of melanocytes within the leptomeninges and/or central nervous system (CNS) parenchyma [1]. To date, no studies have been found that statistically analyze the incidence and mortality of the disease. For instance, one study searched the U.S. National Library of Medicine (NLM) using the keyword “neurocutaneous melanocytosis” and retrieved only 116 relevant results spanning the period from 1976 to 2024 [2]. The pathogenesis of NCM is believed to originate from aberrant migration of neural crest‐derived melanocytes during embryonic development, with subsequent infiltration into the CNS via paraspinal ganglia and peripheral nerve sheaths [3]. Pediatric NCM typically manifests before the age of 2 years and is often associated with congenital melanocytic nevi (CMN). These patients may present with progressive neurological symptoms in the absence of an identifiable precipitating factor. In contrast, adult‐type NCM usually presents after the age of 30 years and may be associated with acquired melanocytic lesions or malignant transformation [4]. Currently, there is no established effective treatment for NCM. The prognosis remains poor, particularly for patients presenting with neurological symptoms. Those treated with radiotherapy and chemotherapy generally show limited clinical response, with most succumbing to disease within 3 years following symptom onset [5]. Palliative interventions may offer transient symptomatic relief but do not significantly alter disease progression.

The diagnostic challenges and therapeutic implications of malignant transformation remain poorly understood in both pediatric and adult NCM. Herein, we report two cases diagnosed at the First Affiliated Hospital of Xinxiang Medical University: one involving a child with severe neurological manifestations and another presenting with brain melanoma in an adult. These cases highlight the heterogeneity of NCM and underscore the need for improved understanding of its natural history, early diagnosis, and targeted therapeutic strategies.

## 2 Case History/Examination

### 2.1 Patients No. 1

**FIGURE 1:**
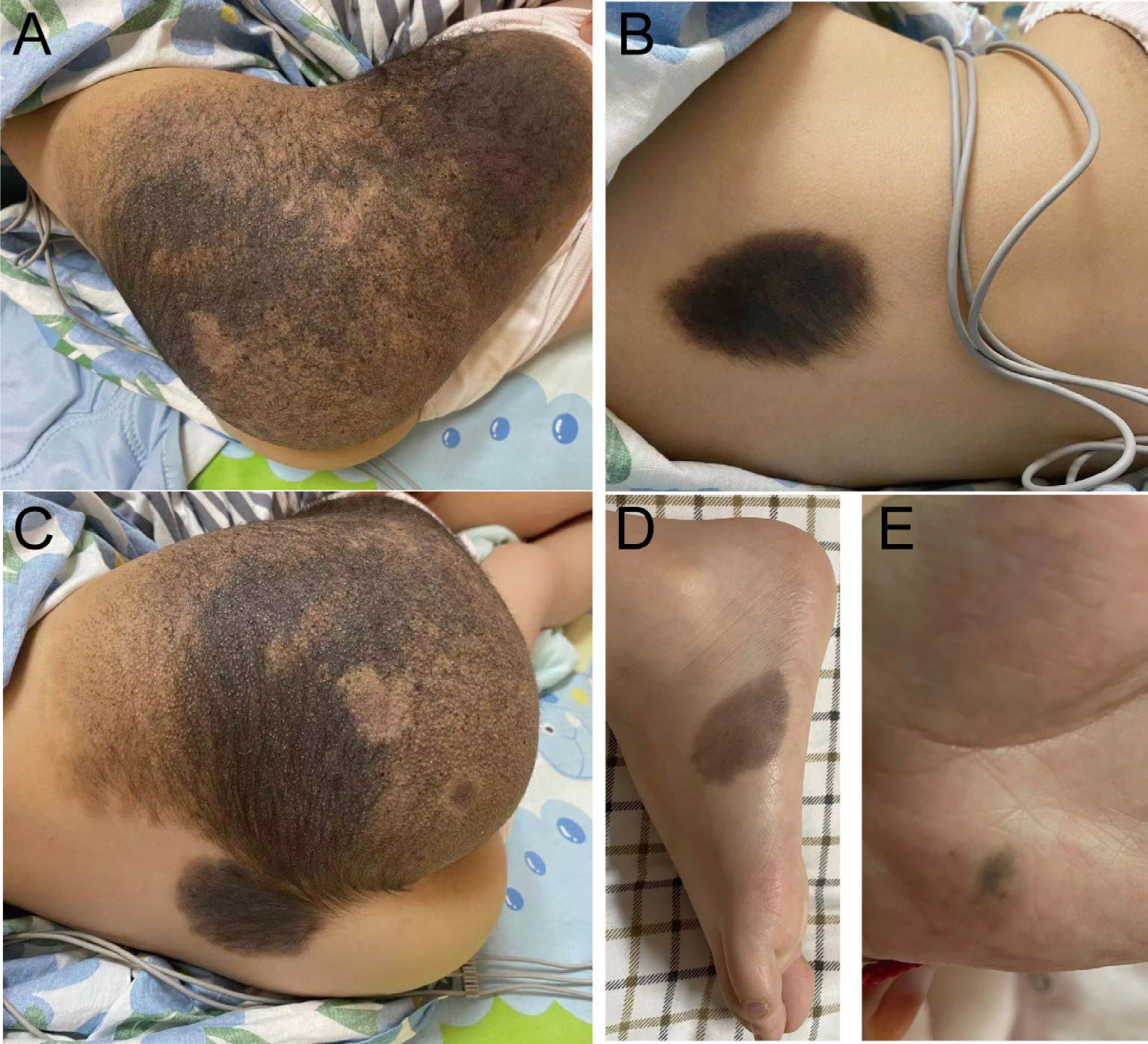
Images of the 6‐year‐old child demonstrating extensive giant melanocytic nevi distributed across multiple body regions.

**FIGURE 2:**
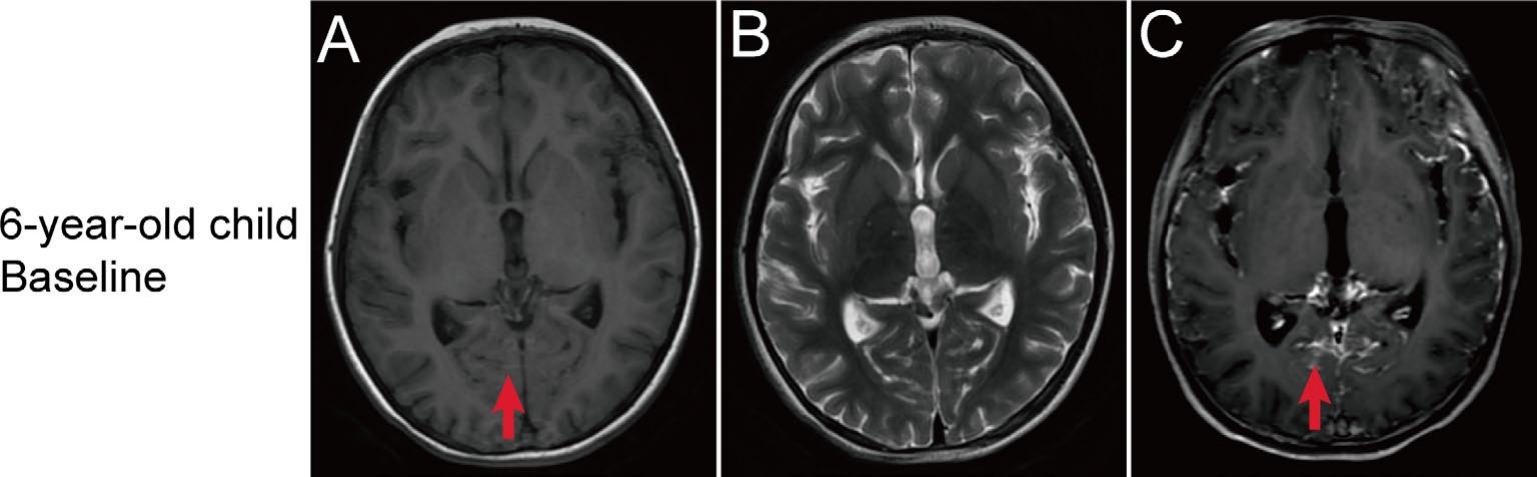
(A) Brain MRI at 6 years of age after the onset of symptoms reveals slightly hyperintense signals in the calcarine sulcus on T1WI. (B) Isointense signals in the calcarine sulcus on T2‐weighted images (T2WI). (C) Enhancement within the cerebral sulci and along the extensive leptomeninges on T1WI.

A young girl aged 6 years and 5 months was born with melanin deposits of varying sizes on the lower back, buttocks, left hand, right thigh, and left foot. The cutaneous nevi grew in size, color, and number as she grew older (Figure 1A–E). At the age of 6 years, she caught a cold and developed a cough and nasal congestion, along with nausea and vomiting. Magnetic resonance imaging (MRI) performed in June 2021 showed hypointense T1‐weighted images (T1WI) and isointense T2 signals seen in the calcarine sulcus and extensive slightly hyperintense signals in the leptomeninges with obvious enhancement (Figure 2A–C). Examination of the cerebrospinal fluid (CSF) revealed no abnormal cells. Then, she was diagnosed with “septic meningitis” and was injected with mannitol to reduce cranial pressure.

**FIGURE 3:**
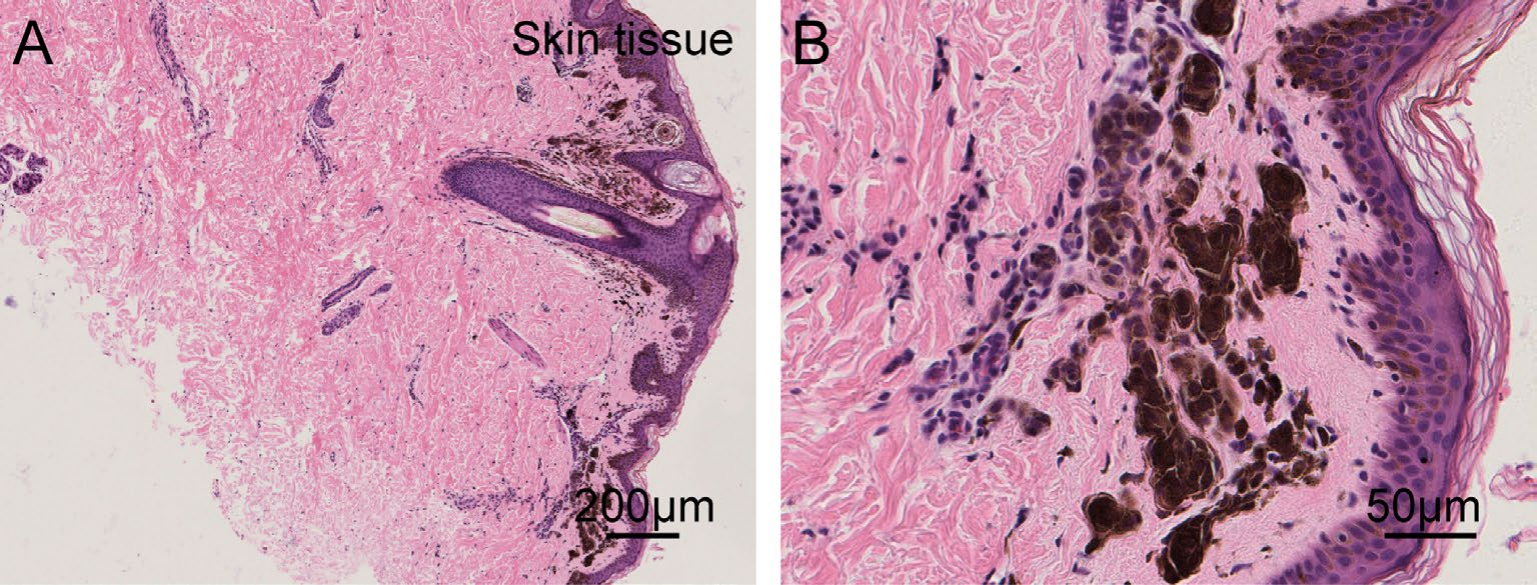
Histopathological examination of 6‐year‐old child skin biopsy specimens from satellite lesions reveals features consistent with benign compound nevus under hematoxylin and eosin (H&E) staining at magnifications of 40× and 200×.

Three months later, the patient developed new symptoms of fatigue, aphasia, and paralysis of the right limb. The MRI revealed a significant high signal in the intracerebral soft meningeal enhancement and a dilated ventricular system with wider sulci and fissures three months later. The CSF cytology results showed a small number of lymphocytes. Over time, a ventriculoperitoneal shunt (VPS) was performed to reduce the intracranial pressure and thus alleviate the severe symptoms of nausea and vomiting. During the surgical procedure, black‐colored brain tissue was observed. A biopsy was not performed because of a rich blood supply to the tumor and the difficulty in controlling the bleeding. To acquire the pathological diagnostics, the microsatellite lesions on the skin of the right lower extremity were examined, and the result indicated a compound nevus in October 2021 (Figure 3A,B).

### 2.2 Patient No. 2

**FIGURE 4:**
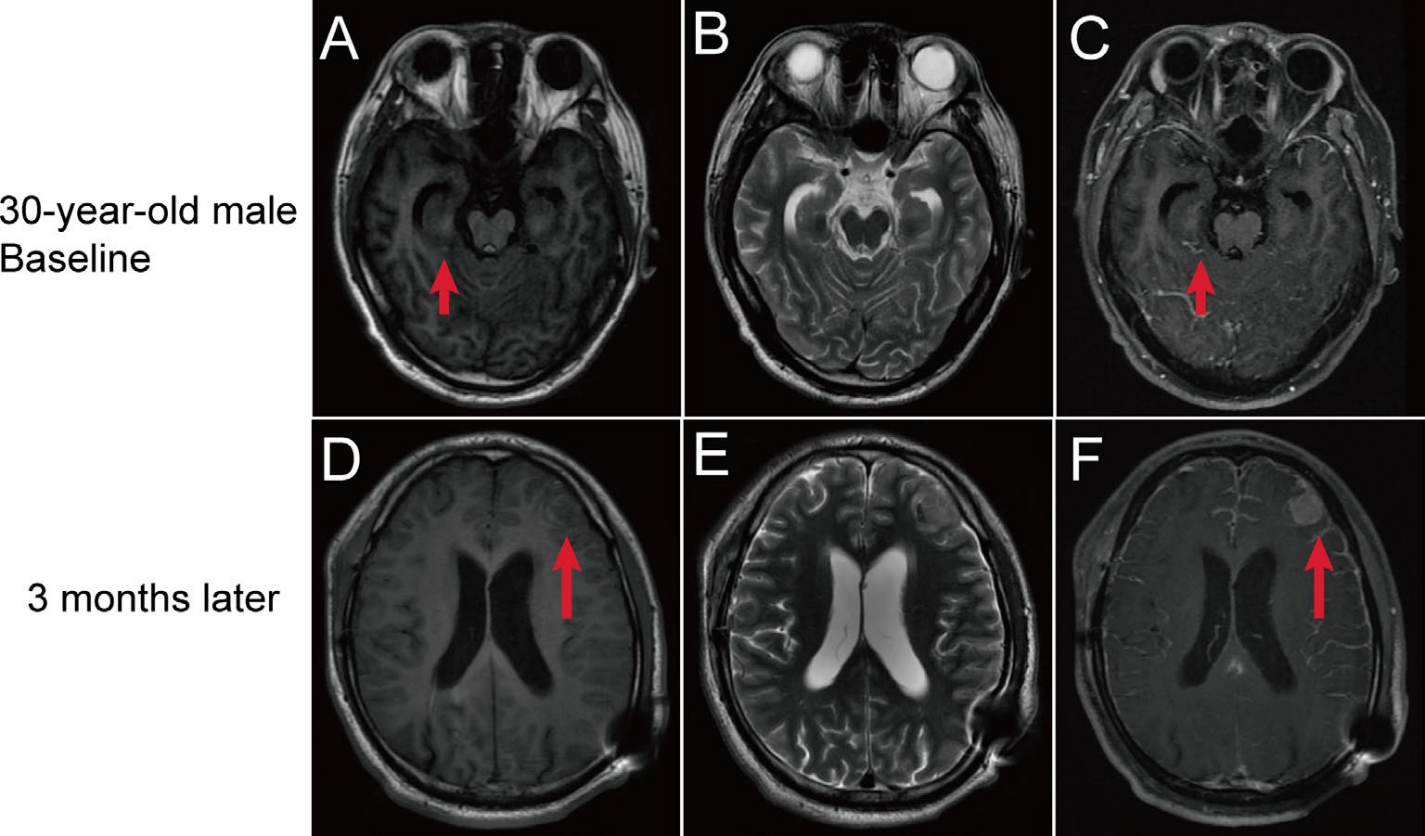
(A–C) Brain MRI at 30 years of age, after the onset of neurological symptoms, shows a patchy region with slightly hypointense T1 signals (A) and slightly hyperintense T2 signals (B) in the uncus of the right parahippocampal gyrus; leptomeningeal enhancement is markedly increased on T1 FLAIR after contrast enhancement (C). (D–F) Follow‐up MRI performed 3 months later reveals a nodular lesion in the left frontal lobe with isointense to slightly hypointense T1 signals (D) and isointense to slightly hyperintense T2 signals (E); the lesion has a well‐defined border and demonstrates prominent, homogeneous enhancement on T1 FLAIR contrast‐enhanced imaging (F).

**FIGURE 5:**
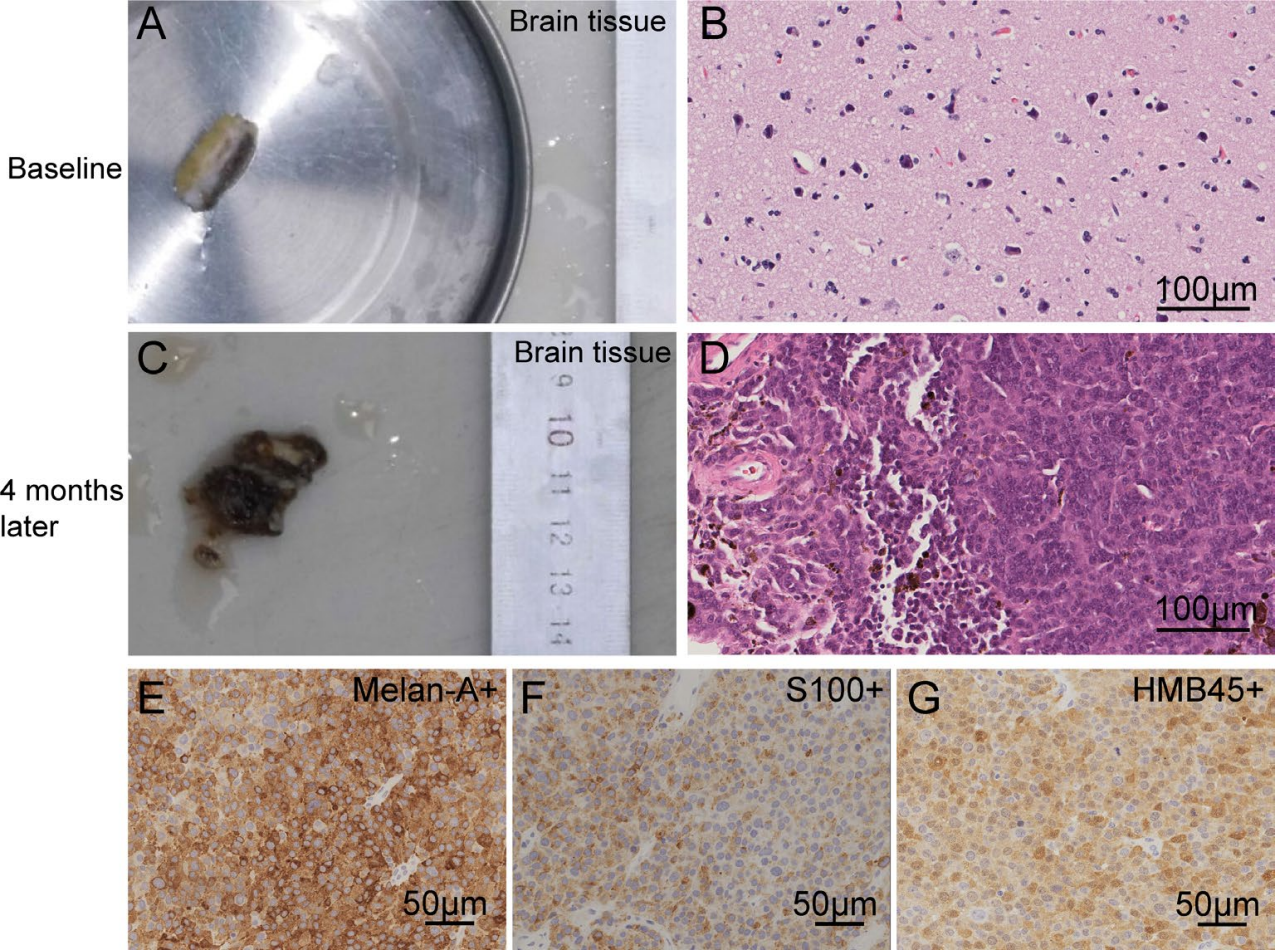
(A, B) Histopathological analysis of brain lesions showed brain tissue with no structural abnormalities and no typical melanocytes at the baseline. (C, D) The pathological results revealed that the cortical dark tissue and left frontal occupying lesion indicated malignant melanoma after 4 months. (E–G) Results of immunohistochemical staining showed positive expression of Melan‐A, S100, and HMB45.

A 30‐year‐old male patient was born with scattered black patches that enlarged gradually. The patient was diagnosed with NCM at the age of 29 due to a headache and was found to have a large congenital melanocytic nevus involving the dorsum of the left hand, left eyebrow arch, dorsum of the left foot, and limb areas, along with multiple satellite lesions. After one year, he was admitted to our hospital with a severe headache, accompanied by blurred vision, fatigue, tinnitus, nausea, vomiting, and a single episode of transient loss of consciousness that resolved spontaneously within 10 min. In November 2020, a brain MRI demonstrated a lamellar region of slightly hypointense T1 and slightly hyperintense T2 signals in the right parahippocampal gyrus. Following contrast administration, there was a marked increase in leptomeningeal enhancement on T1WI, which was particularly prominent in the bilateral frontal regions (Figure 4A–C). He was subjected to a lumbar CSF examination, which showed scattered‐nuclear large heteromorphic cells that were shaped like melanocytes. To further confirm the diagnosis, resection of the intracranial metastases was performed. During the operation, significant gray‐black pigmented deposition was seen in the cerebral cortex, while no obvious tumor nodules were found in the sulci and gyri. The leptomeningeal melanocyte deposits for pathological examination did not reveal any typical melanocytes in the temporal lobe mass (Figure 5A,B). Then, he was treated with a VPS to lower the intracranial pressure and reduce vomiting.

Three months later, the patient was readmitted due to a sudden episode of loss of consciousness, accompanied by limb convulsions, head retraction, left eye upgaze and lateral deviation, elevation of the fingers of the left upper limb, foaming at the mouth, vomiting of food, and symptoms lasting approximately 2 min. In February 2021, a brain MRI showed that the slightly short T1 and long T2 nodular signals were seen in the left frontal lobe and pineal region, with clear borders and a mixed high‐low signal on T1WI after contrast enhancement, with sizes of approximately 17 mm × 14.7 mm × 14 mm and 12 mm × 6.72 mm × 4.5 mm, respectively (Figure 4D–F). After one month, the patient experienced severe nausea and vomiting. Brain CT revealed a mixed‐density lesion in the left frontal lobe, which had a size of about 51.7 mm × 45.6 mm × 43.2 mm.

## 3 Diagnosis and Treatment

In the first patient, the following diagnoses were made, taking into account the medical history and auxiliary examinations: (1) NCM, melanoma could not be excluded; (2) elevated intracranial pressure; (3) malnutrition. VPS was performed, and nutritional support was given. The second patient was diagnosed with melanoma transformed from NCM with elevated intracranial pressure, and the possibility of brain herniation could not be ruled out and subsequently underwent surgical treatment.

## 4 Conclusion and Results

For patient 1, after symptomatic treatment by reduction of the intracranial pressure and nutritional support, the patient's parents refused further treatment. The patient died three months after the follow‐up. For patient 2, acute intracranial hypertension occurred, followed by cerebral herniation, and subsequent percutaneous craniotomy tumor resection coupled with craniocerebral repair was performed by the neurosurgeon. During the operation, the black cerebral cortex was found, and part of the tumor tissue was removed. Pathological results indicated that the cortical dark tissue and left‐frontal‐occupying lesions were malignant melanoma after 4 months (Figure 5C–G). Following surgery, the patient died soon after due to postoperative complications.

## 5 Discussion

NCM is a rare congenital neurocutaneous disorder characterized by the presence of large or giant congenital melanocytic nevi (L/GCMN) on the skin and benign or malignant melanocytic proliferation within the CNS, particularly in the leptomeninges and brain parenchyma [6]. Patients with NCM may present with a wide range of neurological manifestations, including signs of increased intracranial pressure (e.g., headache, nausea, and vomiting), hydrocephalus, seizures, cranial nerve palsies, sensorimotor deficits, and developmental delay [7]. These symptoms typically appear before the age of 2 years, although atypical presentations beyond this age have been documented [1]. Notably, some patients may remain asymptomatic during early infancy, making early diagnosis challenging. Currently, there is no established curative treatment for NCM [8]. The prognosis remains poor, especially in patients who develop neurological symptoms. Early detection and monitoring of CNS involvement are crucial for timely intervention and potential improvement in clinical outcomes. Brain MRI is considered the imaging modality of choice for identifying leptomeningeal and parenchymal melanocytic infiltration, which often appears as abnormal contrast enhancement on T1‐weighted sequences.

In this study, we presented two cases of NCM patients in different age groups. Case 1 was a pediatric patient born with extensive cutaneous melanocytic nevi consistent with L/GCMN. Neurological symptoms emerged at the age of 6 years, later than the typical onset of pediatric‐type NCM. This delayed presentation highlights the importance of regular CNS surveillance using MRI, even in the absence of early neurological signs. Although CSF analysis did not reveal melanocytes, serial brain MRIs demonstrated progressive enhancement in the pons, suggesting subclinical meningeal or parenchymal involvement. Case 2 represented an adult‐type NCM. The patient had a history of widespread cutaneous pigmentation since birth and developed neurological symptoms in adulthood. MRI showed progressive leptomeningeal enhancement and lesions in the left frontal lobe. Histopathological examination following surgical resection confirmed the presence of malignant transformation into melanoma, underscoring the risk of malignancy in long‐standing NCM cases.

Distinguishing between CMN, NCM, and metastatic melanoma (MM) is critical for accurate diagnosis and appropriate management. While CMN is limited to the skin, NCM involves the CNS without evidence of distant metastasis. In contrast, MM typically presents with systemic dissemination and histopathological features of malignancy [9, 10]. It is worth noting that NCM may be more readily detectable in the unmyelinated brain during early development, supporting the recommendation for routine CNS MRI surveillance in children with L/GCMN [11]. However, a subset of patients may show no definitive abnormalities on MRI, emphasizing the need for comprehensive diagnostic approaches, including CSF analysis and histopathological evaluation when indicated. Given the lack of standardized diagnostic criteria, a high index of suspicion remains essential for timely diagnosis and management.

In 1991, Kadonaga and Frieden proposed a set of diagnostic criteria for NCM based on a comprehensive review of the existing literature [12]. According to their definition, the diagnosis of NCM requires the following three key features: (1) The presence of a large congenital melanocytic nevus (greater than 20 cm in diameter) and/or multiple CMNs (> 3 in number), in association with meningeal melanosis or melanoma; (2) Histopathological examination revealing benign melanocytic infiltration in the meningeal lesions, without evidence of cutaneous melanoma involvement; (3) Histologically benign cutaneous melanocytic nevi, with no evidence of meningeal melanoma infiltration. These criteria remain widely referenced in contemporary clinical and pathological evaluations of NCM. In the present report, both pediatric and adult‐type NCM cases exhibited typical clinical and imaging features consistent with the diagnostic framework established by Kadonaga and Frieden. However, it is important to note that these criteria were primarily derived from pediatric cases and may not fully capture the spectrum of adult‐onset NCM, particularly in the context of malignant transformation and atypical presentations.

Symptomatic NCM is associated with a poor prognosis, with approximately 50% of affected children succumbing to the disease by the age of five years. In our clinical observations, Patient 1 survived for only 8 months after symptom onset, while Patient 2 survived for 13 months. Currently, there are no established effective therapeutic strategies for NCM, and management remains largely palliative [13]. Patient 1 received supportive care, including intracranial pressure reduction and nutritional support, but did not undergo any anti‐tumor therapy despite histopathological evidence of melanoma transformation. This highlights a critical challenge in clinical decision‐making: during the early stages of NCM, patients may remain histologically benign for extended periods despite experiencing severe neurological symptoms. Therefore, establishing standardized treatment guidelines for both early‐stage NCM and following malignant transformation is urgently needed [14]. Patient 2 was initially diagnosed with NCM without clear evidence of melanoma transformation in the brain. One year later, the patient developed extensive tumor involvement of both the leptomeninges and brain parenchyma. Despite surgical resection, rapid tumor progression, postoperative complications, and recurrence ultimately led to a fatal outcome. Therefore, whether there are better therapeutic strategies for early intervention may improve clinical outcomes and prolong survival.

There is no specific or curative treatment for NCM, and therapeutic strategies are largely limited to palliative interventions, including surgical resection, radiotherapy, and chemotherapy. In cases of malignant transformation into melanoma, more aggressive multimodal therapies may be considered; however, clinical outcomes remain poor, and no definitive cure has been established for NCM. Emerging evidence suggests that molecular alterations may play a critical role in the pathogenesis of NCM. For instance, Takayama et al. reported higher levels of MET protein, a cellular receptor for HGF, in lesional tissue from an NCM patient [15]. However, this case did not investigate the presence of oncogenic mutations in *NRAS* or *BRAF*, which are commonly implicated in melanocytic tumors. Subsequent case reports have identified *NRAS Q61K/R* or *BRAF* mutations in NCM patients, but did not assess MET protein levels in corresponding tissues [2]. These findings highlight the molecular heterogeneity of NCM and underscore the need for comprehensive molecular profiling to guide targeted therapy.

Although case reports on NCM remain rare, they provide valuable insights into potential therapeutic approaches and their impact on disease regression and survival. A case series described four patients with NRAS‐mutant CNS melanoma associated with CMN and neurologic abnormalities who were treated with trametinib. All patients showed mild clinical improvement, with symptom‐free periods ranging from one to nine months; however, the disease rapidly progressed after treatment discontinuation, leading to death [16]. Improved outcomes have been observed when trametinib is used in combination with other agents. In 2020, Vanood et al. reported on a 13‐year‐old NCM patient with multiple CMN and seizure onset. Genetic testing revealed the NRAS Q61K mutation, and MRI confirmed leptomeningeal involvement. Initial seizure control was achieved with levetiracetam, later escalated, and combined with trametinib. At 10‐month and 1‐year follow‐ups, the patient remained seizure‐free with no neurological progression [17], suggesting that early intervention may improve prognosis. These cases indicate that genetic heterogeneity significantly influences treatment response. Pre‐treatment molecular profiling may therefore guide personalized therapy. However, current outcomes remain unsatisfactory, highlighting the need for further research into the pathogenesis of NCM and the development of more effective targeted therapies.

## Author Contributions

**Yinghua Ji:** resources, writing – original draft, writing – review and editing. **Keya Zhi:** writing – original draft, writing – review and editing. **Han Yang:** data curation, writing – review and editing. **Xiangli Meng:** writing – original draft, writing – review and editing. **Jin Wang:** writing – original draft, writing – review and editing. **Jinling Xie:** writing – original draft, writing – review and editing. **Yong Tan:** writing – original draft, writing – review and editing. **Ping Lu:** writing – original draft, writing – review and editing. **Yana Zhang:** conceptualization, writing – original draft, writing – review and editing.

## Ethics Statement

The study received approval from the Institutional Ethics Committee of the First Affiliated Hospital of Xinxiang Medical University.

## Consent

The patient provided written informed consent.

## Conflicts of Interest

The authors declare no conflicts of interest.

## Data Availability

The data are available from the corresponding author on reasonable request.
